# Synthesis of a novel PEGylated colon-specific azo-based 4- aminosalicylic acid prodrug

**DOI:** 10.22038/ijbms.2020.41152.9736

**Published:** 2020-06

**Authors:** Fatemeh Sadeghi, Atie Eidizade, Farinaz Saremnejad, Farzin Hadizadeh, Elham Khodaverdi, Abbas Akhgari

**Affiliations:** 1 Targeted Drug Delivery Research Center, Pharmaceutical Technology Institute, Mashhad University of Medical Sciences, Mashhad, Iran; 2 Department of Pharmaceutics, School of Pharmacy, Mashhad University of Medical Sciences, Mashhad, Iran; 3 Department of Food Science and Technology, Ferdowsi University of Mashhad, Mashhad, Iran; 4 Biotechnology Research Center, Pharmaceutical Technology Institute, Mashhad University of Medical Sciences, Mashhad, Iran; 5 Department of Medicinal Chemistry, School of Pharmacy, Mashhad University of Medical Sciences, Mashhad, Iran

**Keywords:** 4-aminosalicylic acid, Azo linkage, IBD, PEGylated prodrug, Prodrug

## Abstract

**Objective(s):**

4-aminosalicylic acid (4-ASA) is an isomer of mesalazine that has recently been shown to be effective against inflammatory bowel disease (IBD), and more specifically, ulcerative colitis. However, the majority of orally administered 4-ASA is readily and extensively absorbed from the stomach and small intestine, so only a small amount is transported to the colon. A mutual ester and azo prodrug of 4-ASA was synthesized with polyethylene glycol (PEG) and dimethylaniline, respectively , to overcome this issue.

**Materials and Methods:**

The 4-ASA prodrug was synthesized via a two-step process and then characterized by 1H-NMR. The stability of the prodrug was evaluated in simulated gastric fluid (pH 1.2). Furthermore, the *in vitro* release profiles of the drug conjugate was evaluated at pH 1.2, as well as pH 6.8 in the absence or presence of rat cecal content.

**Results:**

The prepared prodrug was stable at pH 1.2, indicating that it could be protected from the acidic environment of the stomach. Also, the results of drug release at pH 6.8 showed that the amount of 4-ASA released was 63% within 12 hr in the absence of rat cecal content, while in the presence of rat cecal content, 97% of 4-ASA was released from the prodrug in 6 hr.

**Conclusion:**

Overall, the synthesized PEGylated azo-based 4-ASA prodrug could be a potential candidate for targeted drug delivery to the inflamed gut tissue in IBD.

## Introduction

Colon targeted drug delivery systems have been used successfully in the treatment of disorders in the colonic region, such as Crohn’s diseases (CD), ulcerative colitis (UC), and colon cancer (1–3). CD and UC are the two primary forms of inflammatory bowel disease (IBD). CD affects any part of the gastrointestinal tract (GIT), whereas UC is restricted to the colon and rectum (4–8). Induction and maintenance of remission, mucosal healing, the avoidance of surgical intervention, and decreasing the likelihood of cancer development are the primary therapeutic goals in UC (9–12).

Aminosalicylates are first-line therapies in the treatment of IBD (13). Mesalamine (5-aminosalicylic acid, 5-ASA) is considered to be the most efficient of the aminosalicylates and is consequently widely prescribed. 5-ASA is effective in IBD treatment and prevention of its recurrences. This drug is usually a well-tolerated option; however, various side effects that are associated with its administration include skin, hematological, hepatic, cardiac, pulmonary, digestive, and renal disorders, and also acute edematous pancreatitis of immune-allergic origin (14). 

**Figure 1:**
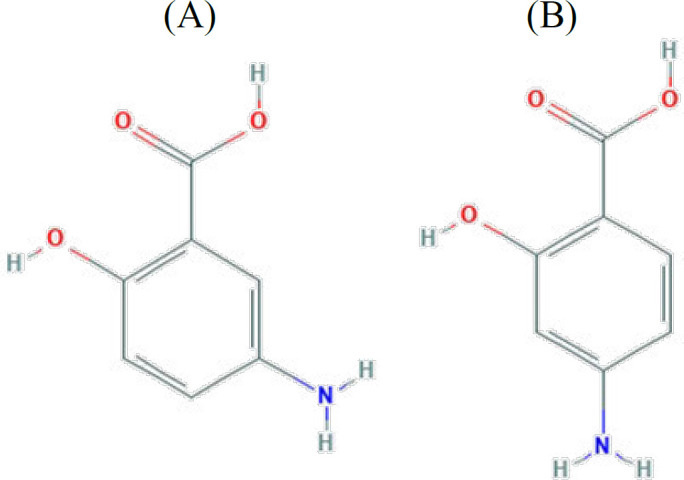
Chemical structures of 5-aminosalicylic acid (A) and 4-aminosalicylic acid (B)

Recently some shift from 5-ASA to 4-ASA (4-aminosalicylic acid, a second line anti-tuberculosis agent) has been reported in the treatment of IBD by introducing a number of 4-ASA prodrugs in the literature (15). 4-ASA is the isomer of 5-ASA, and its chemical structure is only distinguished from 5-ASA by the position of its NH_2_ group (Figure 1), though this small variation is sufficient to affect all of the biological activities of the isomers, their melting points, solubility, and molecular weights are otherwise identical (153.13 g/mol) (16) to 5-aminosalicylic acid (5-ASA, mesalazine.

Research studies showed that apart from significant anti-tuberculosis activity, 4-ASA provides a stable, effective and inexpensive alternative to 5-ASA for the topical treatment of both active and quiescent UC with lesser side effects, and also has been designated an orphan drug by the FDA for mild to moderate UC (17–19). 

To achieve the successful delivery of drugs to the colon, the drug needs to be protected from the GIT environment or absorption in the upper GIT and be released in the target site (20). There are mainly two types of approach to deliver a drug to the colon: (I) delivery of the intact drug to the colon via manipulation of its dosage form; (II) formation of covalent linkage between drug and a carrier with the potential of digestion in the colon. The delivery of an intact form of the drug includes coating with pH-sensitive polymers, biodegradable polymers, redox-sensitive polymers, embedding in biodegradable matrices and hydrogels, etc. The prodrug approach is commonly believed to be one of the most effective methods for the targeted delivery of drug to the colon, in which drug is covalently bound to the carrier. The covalent linkage of the drug to a carrier could be via different types of bonds and lead to formation of different conjugates such as azo conjugates, glycoside conjugates, glucuronide conjugates, cyclodextrin conjugates, amino acid conjugates, and polymeric conjugates (20–22).

Prodrug could improve the physicochemical properties and drug delivery by increasing the drug concentration at the target site and decreasing its toxicity and undesirable side effects (1). Various polymeric prodrugs have been designed for targeting the drug to the colon, where the drug is covalently linked to a polymeric promoiety. Biodegradable polymers such as polylactides (PLA), polyglycolides (PGA), poly (lactide-co-glycolides) (PLGA), polyanhydrides, polyorthoesters, poly (2-hydroxyethyl methacrylate), poly (methyl methacrylate), poly (vinyl alcohol), polyacrylamide, poly (ethylene glycol), poly (methacrylic acid), chitosan, guar gum, dextran, cyclodextrins, polyphosphazene, etc., have been widely used in this area (23).

This study aimed to protect the orally administered 4-ASA against absorption from the stomach and small intestine. This idea is based on the synthesis of a mutual ester and azo prodrug of 4-ASA, which could be a potential candidate for targeted drug delivery to the inflamed gut tissue in IBD.

## Materials and Methods

***Materials***

4-aminosalicylic acid was purchased from ACROS Organics™, US. Sodium nitrite, hydrochloric acid, N, N-dimethylaniline, acetic acid, sodium acetate, polyethylene glycol 6000, N, N-(dimethylamino) pyridine, dimethylformamide and N, N-dicyclohexylcarbodiimide were supplied from Merck, Germany. All chemicals used in the synthesis were of AR grade.

***Methods***

*Synthesis of the azo compound*

**Figure 2:**
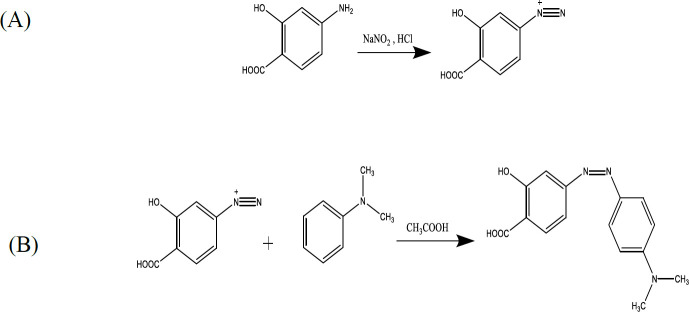
4-aminosalicylic acid reaction with dimethylaniline and synthesis of the azo compound

1 g (6.53 mmol) of 4-ASA was transferred into an Erlenmeyer flask and dissolved in about 30 ml distilled water with the aid of heat (below 50 ^°^C). After cooling the solution to room temperature (about 25 ^°^C), 0.4 g (5.8 mmol) of sodium nitrite, which was dissolved in 1 ml of water was added to the flask. The content of the flask was added slowly to a large beaker containing 6 g of crushed ice and 1.1 ml (6.6 moles) of concentrated hydrochloric acid (6 molars) while stirring (Figure 2-A). Then the beaker was placed in the refrigerator and left for 15 min to allow for precipitation of diazonium salt. A mixture of 0.7 ml of N, N-dimethyl aniline (5.5 mmol) and 0.4 ml of concentrated acetic acid was added slowly to the diazonium salt and stirred for 30 min to complete the reaction (Figure 2-B). The amount of 0.55 g (6.6 mmol) of sodium acetate solution was added to the content of the beaker, and after a few minutes, the precipitate was filtered. The red precipitate was washed with cold water and allowed to dry for one day at room temperature. 

*PEGylation of Azo compound*

**Figure 3:**
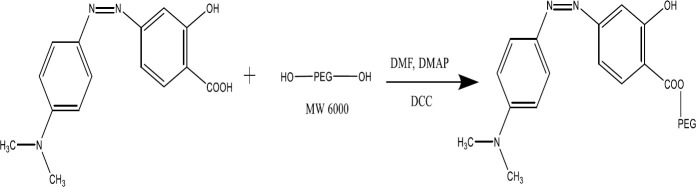
Azo reaction with PEG and synthesis of PEG-Azo

DMF: dimethylformamide; DMAP: N,N-dimethylamino pyridine; DCC: N, N-dicyclohexyl carbodiimide

0.1 g (0.36 mM) of the azo product, 0.044 g (0.36 mmol) of N, N-dimethylamino pyridine (DMAP) and 0.074 g (0.36 mmol) of N, N-dicyclohexyl carbodiimide (DCC) were transferred into 5 ml of dimethylformamide (DMF) and placed on a stirrer for 30 min (Figure 3). Then, 1.8 g (0.3 mM) of polyethylene glycol 6000 was added and stirred for 6 hr. The resulting dicyclo-hexyl-urea (DCU) precipitate was filtered. The clear solution obtained was transferred to a Petri dish and placed at room temperature for 24 hr to dry. The red powder obtained from the Petri dish was collected (24).

*Prodrug purification*

The synthetic prodrug was dissolved in a small amount of distilled water and transferred into a pretreated dialysis bag with cut-off 500-1000 Dalton and was knotted at the end. The dialysis bag was placed in a beaker containing distilled water. After several hours, the content of the dialysis bag was poured into a Petri dish and left for one day at room temperature to become dry. By this purification method, the synthesized prodrug remained in the dialysis bag.

*Characterization of the synthesized compounds by* ^1^ *H nuclear magnetic resonance*

The ^1^H-NMR spectra were taken on a Brucker spectrometer (Bruker Avance, Germany) at 300 MHz proton resonance frequency.

*Calibration curves of drug and synthesized prodrug*

For the analysis of drug and synthesized prodrug by UV spectrophotometry, standard calibration curves were constructed by measuring the absorbance of the standard solutions in various media at respective wavelengths according to Table 1.

*Stability of prodrug*

The stability of the prodrug was evaluated at pH 1.2. The amount of 10 mg of prodrug was dissolved in a small amount (2-3 ml) of the relevant medium and was transferred into the dialysis bag. The dialysis bag was placed in 50 ml of each medium separately and stirred continuously at 37 ^°^C. At different time intervals, the absorbance of 4-ASA at related λ_max_ was measured for each media by a spectrophotometer. Then, according to the standard calibration curves, the concentration of the drug was calculated with three repetitions. 

*In vitro dissolution studies*

140 mg of prodrug (containing 3.45 mg of 4-ASA) was accurately weighed and dissolved in a small amount (2-3 ml) of the relevant medium and was then transferred into the dialysis bag. The dialysis bag was knotted at the end and was placed in a 150 ml HCl 0.1N with pH 1.2 at 37 ^°^C and was continuously stirred. At specified time intervals for 2 hr, 5 ml samples were taken and replaced with 5 ml of the same medium. The same procedure was repeated in phosphate buffer pH 6.8 for 12 hr (each experiment was repeated three times). A similar test was performed in phosphate buffer pH 6.8 containing 4% w/v rat cecal contents under the flow of CO_2_ to simulate the anaerobic condition in the colon. 140 mg of prodrug was dissolved in 2 ml of the above medium and transferred into the dialysis. The dialysis bag was clamped and placed in a 150 ml phosphate buffer solution pH 6.8, at a temperature of 37 °C. The medium was continuously stirred. At the specified time intervals, 5 ml of the sample was removed for 12 hr and was replaced with 5 ml of phosphate buffer solution pH 6.8 (each experiment repeated three times). The absorbance of the samples at λ_max_ of 4-ASA was measured using a spectrophotometer, and the amount of 4-ASA released in different media was calculated based on the standard curves that were previously obtained (2).

## Results

***Characterization of the synthesized compounds by 1H-NMR***

The results of the HNMR of the prodrug in both synthesis steps are presented below. Results showed that in both steps, the reaction was performed and the desired combination was synthesized. The first and second steps were performed with a yield of 33.33% and 68%, respectively.

*Azo compound*

^1^HNMR (300MHz, DMSO-d_6_, δ, ppm): 8.6 (s, 1H, OH), 8.0-6.7 (4d, s , 7H, Ph-H_5’,6’, 2, 3, 5, 6 _ & Ph-H_2’_), 3.0 (s, 6H, N(CH_3_)_2 _)

*PEG-Azo compound*

^1^HNMR (300MHz, DMSO-d_6_, δ, ppm): 8.6 (s, 1H, OH), 8.0-6.7 (4d, s , 7H, Ph-H_5’,6’, 2, 3, 5, 6 _ & Ph-H_2’_), 3.5(s, PEG6000-H), 3.0 (s, 6H, N(CH_3_)_2 _).

***Stability of prodrug***

The stability of the prodrug was evaluated, and related values are indicated in Table 2. According to these results, the amount of 4-ASA release was very low after 120 min at pH 1.2, i.e., more than 93% of prodrug remained intact at pH 1.2, indicating prodrug stability at these media.

***In vitro*** ***dissolution studies***

According to the results of the release test, the amount of drug released during the sampling time was calculated. The following graphs show the amount of drug released versus time in different media.

**Figure 4.**
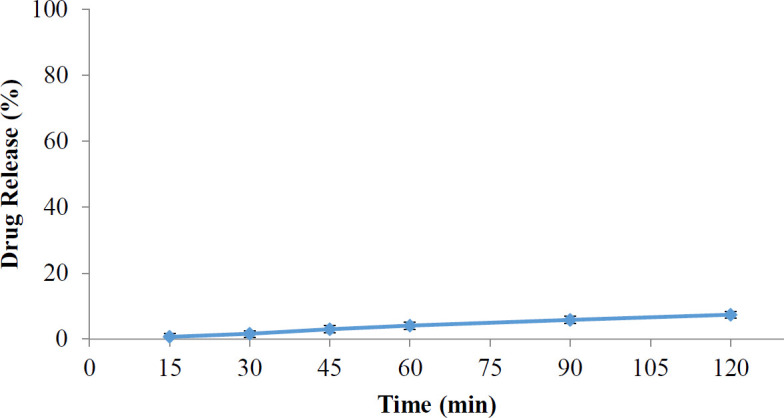
4-aminosalicylic acid release profiles at pH=1.2

**Figure 5:**
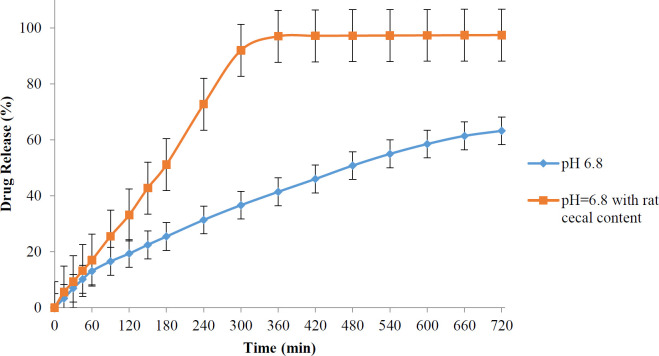
4-aminosalicylic acid release profiles in phosphate buffer pH=6.8 in presence or absence of rat cecal content

As can be seen in Figure 4, the 4-ASA released from the synthetic prodrug at pH 1.2 after 2 hr was about 5.7%, which is almost consistent with the results of the prodrug stability test in this medium. The amount of drug release after 6 hr in phosphate buffer pH 6.8 was approximately 40% in the absence of rat cecal content. In contrast, this amount was increased to 97% in the presence of rat cecal content, and almost all of 4-ASA was released from the prodrug (Figure 5).

## Discussion

Oral drug delivery to the colon, especially with the goal of effective treatment of IBD, is important. 5-ASA is an anti-inflammatory drug used in the treatment of IBD. 5-ASA delivery to the colon by conventional dosage forms and via the oral route has some limitations. This drug is absorbed mainly in the upper gastrointestinal tract, and a low amount of drug is able to reach the colon. Hence the design of a colon targeted drug delivery system with the focus on delivering a large portion of the drug to the colon and preventing its release in the upper gastrointestinal tract can be an excellent way to increase the effectiveness and reduce the side effects of 5-ASA. For this reason, so many efforts have been made, and many formulations for 5-ASA as modified release products and prodrugs have been suggested (25). Several prodrugs of 5-ASA have been formulated for drug-targeted delivery to the colon, including azo-prodrugs (26), ester-prodrugs (27), polysaccharide-prodrugs (28), amide-prodrugs (29), and polymeric prodrugs (30).

4-ASA is a structural isomer of the 5-ASA, which, according to some studies, is significantly more potent and effective in the treatment of IBD than 5-ASA (16). It also has fewer side effects. For example, acute pancreatitis, which is sometimes observed following 5-ASA administration, is not observed with 4-ASA. Therefore, 4-ASA could be a selective drug for the treatment of IBD in patients with pancreatitis. This drug has also been formulated as modified release products and prodrugs with the goal of drug delivery to the colon with increased efficacy.

Various prodrugs of 4-ASA that have been studied include glycoside- prodrugs, amide-prodrugs, azo-prodrugs, and steroid-prodrugs. It has been reported that these prodrugs have shown better efficacy than 4-ASA and 5-ASA (15).

In this study, the synthesis of a mutual prodrug of 4-ASA with an ester bond with PEG polymer and an azo bond with N, N-dimethyl aniline, was investigated.

**Figure 6:**
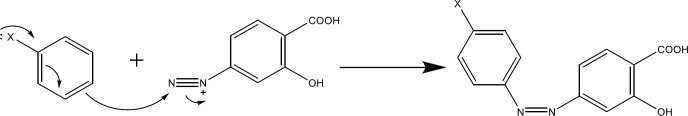
Synthesis of the prodrug with the azo bond in the presence of a group (X) with electron donor properties

In the first step of the synthesis of the prodrug, a prodrug with an azo bond was formed. N, N-dimethyl aniline was used as an inactive moiety for the reaction with 4-ASA and formation of the azo bond. N, N-dimethyl aniline is a suitable electron donor and can react and form an azo bond with 4-ASA (Figure 6). 

The synthesized azo compound was salt and therefore was soluble in the acidic and alkaline environment. The first reaction was carried out in an acidic environment, and therefore, a portion of the azo compound was present as a soluble form in the reaction environment. By adding sodium acetate solution and changing the acidic pH to the isoelectric pH, the azo compound was precipitated entirely, and the synthesized azo product was isolated. Also, as the azo compounds are colored, the red color of the synthetic azo compound has further confirmed the occurrence of reaction and formation of the product. The synthesized azo product was also identified and confirmed by the interpretation of its HNMR spectrum. 

In the second stage of the prodrug synthesis, PEG was coupled with high yield to the azo compound. The PEG was used to prevent the absorption of the prodrug in upper parts of GIT via its large molecular size and steric hindrance. PEG in the HNMR spectrum appeared as a distinct peak in the region of 3.5, and the PEG-Azo compound was identified.

A dialysis bag with cut-off 1000 Dalton was used to purify the synthetic product. Due to the large size of the PEG-Azo, this compound was able to remain in the dialysis bag, while the starting material, 4-ASA, due to its small size, was removed from the dialysis bag during the process. 

A stability test of prodrug was performed in a simulated gastric medium with pH 1.2 to make sure that the drug does not release in the upper gastrointestinal tract.

Previously in stability studies on 5-ASA prodrugs, it has been reported that some prodrugs were almost stable in the upper gastrointestinal environments. For example, it was shown that an amide prodrug of 5-ASA has been stable in a simulated gastric environment (31). 

In another study, it was reported that the concentration of drug in the stomach and small intestine was less when cyclodextrin prodrug of 5-ASA was used compared to when the free drug was used (32). Dendrimer formulations were also designed and tested in medium with pH 1.2 and in homogenates of gastric tissues of rats, and almost no free drug was found in these media (33).

Stability studies on 4-ASA prodrugs have also demonstrated the stability of prodrugs in the upper gastrointestinal tract. The stability studies on 4-ASA prodrug with amide bond in buffer with pH 1.2 indicated the stability of this type of prodrug in this buffers over 10 hr (25). 

The glycosylated type prodrug of 4-ASA also showed stability in buffers with pH 1.2 over a 12 hr period (34). Also, the stability of the prodrug of 4-ASA and dextran or cyclodextrin with ester-bond was tested. The amount of drug release in buffers with pH 1.2 and the stomach and small intestine homogenates of mice was about 20–23%, which indicated minor hydrolysis in the upper gastrointestinal tract (6).

 Azo prodrugs have also shown stability in the stomach and small intestine and had the least degree of drug release (35, 36). 

In the stability test of the synthesized prodrug which had both azo and an ester bond, the amount of the drug in the simulated gastric medium at pH 1.2 after 2 hr was about 7%. Therefore in comparison with other studies, this prodrug showed acceptable stability in the upper gastrointestinal tract.

The release test was performed for synthetic prodrug to investigate drug release profiles at different conditions and also to evaluate the release of drug at the end part of the digestive tract.

Studies on the release of various 5-ASA prodrugs have shown that they were able to direct drug release in the colon, and thus they could increase the efficacy of the drug (37, 38). Evaluating the azo prodrug of 5-ASA in rat cecal content has shown that this prodrug was susceptible to bacterial enzymes in the colon and mainly released the drug at the end part of the intestine (39). Amide prodrug could also be delivered to the colon with a high amount, where it was broken down by enzymes (31). In a study on ester-prodrug, the drug was also carried mainly to the terminal regions of the digestive tract (32). 

The release rate of various 4-ASA prodrugs in the upper gastrointestinal tract was evaluated. Better efficacy of these prodrugs was observed in comparison with 4-ASA and 5-ASA alone. For amide prodrugs, the drug release in mouse feces was about 86–91% during 20 hr. It was claimed that this prodrug had a better safety profile and less possible harmful effects on the pancreas and liver (25). The release of glycosylated prodrugs was also found to be satisfactory in colon and cecum contents of either healthy or colitis rats and had a better therapeutic effect than the drug alone (34). Also, the release of ester prodrug ester-prodrug in the content of cecum and stool was 67–98%. In other words, with partial hydrolysis in the upper gastrointestinal tract, much of the drug had reached and released in the terminal parts of the intestine. This prodrug also showed a better safety profile on the liver and pancreas (6). It has also been observed that drug release from azo prodrugs of 4-ASA has been mainly in the contents of the cecum (98-96%) (35) or in mouse feces (68-91%). Also, no gastrointestinal toxicity or hepatotoxicity was observed with this azo-prodrug (36).

The synthetic prodrug in this study also showed acceptable results in the release tests. This prodrug showed a limited release in the upper gastrointestinal tract and was able to be delivered almost intactly to the end part of the gastrointestinal tract. The release rate of 4-ASA from prodrug in a medium simulating distal part of the gastrointestinal tract and in the absence of rat cecal content (and therefore the lack of azo-reductase and esterase enzymes) was about 63% in 12 hr, while in a medium, with the rat cecal content it was estimated to be 97% after 6 hr. These results indicate that the synthetic prodrug was able to reach the site of its action (the end part of the small intestine and the colon) and could release its active part in these regions. This prodrug was sensitive to enzymes, and its hydrolysis could be facilitated by the presence of rat cecal content, which makes it suitable for the treatment of colon inflammatory diseases.

**Table 1:** λ_max_ of drug and prodrug in various media

Solutions	λ_max_ (nm)	
	pH 1.2	pH 6.8
4-ASA	300	265
Prodrug	279	278

**Table 2:** The percentage of 4-aminosalicylic acid detected at pH = 1.2 instability study at different time intervals

**Time (min)**	**Percent of 4-ASA detected**
15	0.423
30	1.059
45	2.754
60	4.026
90	5.298
120	6.993

## Conclusion

In this study, a prodrug of 4-ASA, which according to several studies, is significantly more potent, with fewer side effects than 5-ASA was synthesized and was studied *in vitro* with the goal of targeted drug delivery to the colon. In the synthesized prodrug, 4-ASA has bound to N, N-dimethyl aniline via azo bond from one end, and to PEG via ester bond form the other end. PEG was coupled to the azo prodrug to limit the absorption and hydrolysis of the prodrug in the upper section of GIT. The prodrug was stable in the upper gastrointestinal tract (stomach and small intestine) and could be delivered to the end parts of the gastrointestinal tract. The release rate of the active component (4-ASA) increased in the lower parts of the digestive tract, especially in the presence of rat cecal contents, which had the azoreductase and esterase enzymes. Further studies are still required to evaluate the synthesized prodrug *in vivo*.
